# Isolation and Characterization of 1-Hydroxy-2,6,6-trimethyl-4-oxo-2-cyclohexene-1-acetic Acid, a Metabolite in Bacterial Transformation of Abscisic Acid

**DOI:** 10.3390/biom12101508

**Published:** 2022-10-18

**Authors:** Oleg S. Yuzikhin, Alexander I. Shaposhnikov, Tatyana A. Konnova, Darya S. Syrova, Hamza Hamo, Taras S. Ermekkaliev, Valerii P. Shevchenko, Konstantin V. Shevchenko, Natalia E. Gogoleva, Anton A. Nizhnikov, Vera I. Safronova, Alexander A. Kamnev, Andrey A. Belimov, Yuri V. Gogolev

**Affiliations:** 1 All-Russia Research Institute for Agricultural Microbiology, Podbelskogo sh. 3, Pushkin, 196608 Saint Petersburg, Russia; 2 All-Russian Research Institute of Plant Protection, Podbelskogo sh. 3, Pushkin, 196608 Saint Petersburg, Russia; 3 Kazan Institute of Biochemistry and Biophysics, Federal Research Center “Kazan Scientific Center of the Russian Academy of Sciences”, 420111 Kazan, Russia; 4 Institute of Fundamental Medicine and Biology, Kazan (Volga Region) Federal University, Kremlevskaya Street 18, 420008 Kazan, Russia; 5 Institute of Molecular Genetics, Russian Academy of Sciences, Akademika Kurchatova Square 2, 123182 Moscow, Russia; 6 Faculty of Biology, Saint Petersburg State University, University Embankment 7/9, 199034 Saint Petersburg, Russia; 7 Institute of Biochemistry and Physiology of Plants and Microorganisms, Subdivision of the Federal State Budgetary Research Institution Saratov Federal Scientific Center of the Russian Academy of Sciences, 410049 Saratov, Russia

**Keywords:** abscisic acid, microbial metabolite, 1-hydroxy-2,6,6-trimethyl-4-oxo-2-cyclohexene-1-acetic acid, NMR spectrometry, phytohormones, rhizosphere, rhodococcal acid, *Rhodococcus*

## Abstract

We report the discovery of a new abscisic acid (ABA) metabolite, found in the course of a mass spectrometric study of ABA metabolism by the rhizosphere bacterium *Rhodococcus* sp. P1Y. Analogue of (+)-ABA, enriched in tritium in the cyclohexene moiety, was fed in bacterial cells, and extracts containing radioactive metabolites were purified and analyzed to determine their structure. We obtained mass spectral fragmentation patterns and nuclear magnetic resonance spectra of a new metabolite of ABA identified as 1-hydroxy-2,6,6-trimethyl-4-oxo-2-cyclohexene-1-acetic acid, which we named rhodococcal acid (RA) and characterized using several other techniques. This metabolite is the second bacterial ABA degradation product in addition to dehydrovomifoliol that we described earlier. Taken together, these data reveal an unknown ABA catabolic pathway that begins with side chain disassembly, as opposed to the conversion of the cyclohexene moiety in plants. The role of ABA-utilizing bacteria in interactions with other microorganisms and plants is also discussed.

## 1. Introduction

Abscisic acid (ABA) is a versatile isoprenoid signaling molecule produced by a wide range of organisms. It is best studied as an important phytohormone regulating plant growth, development and stress response [1]. The main processes controlled by ABA in plants are the acceleration of abscission, induction of dormancy, inhibition of rooting, and stimulation of stomatal closure [2,3,4]. Significant amounts of ABA and the products of its catabolism are constantly introduced into soil via root exudation, decomposition of abscised shoot tissues and root turnover. It has been shown that ABA transporters located in root epidermal cells maintain efflux of ABA from plant tissues, due to which its concentration in the soil solution gradually increases during the growing season [5]. According to the calculations, ABA concentrations in the soil solution ranged from 0.6 to 2.8 nM [6] and could reach 40 nM in the rhizosphere [7]. It was proposed that not only plants but also ABA-producing microorganisms contribute to the accumulation of ABA in the soil [7].

Abscisic acid in the rhizosphere in combination with cytokinins and auxin can influence the development of both the root and shoot [8]. Early studies have shown that exogenously applied ABA is rapidly taken up by the embryo even through the intact seed coat [9]. While the exogenous ABA does not interfere with the initial germination processes, it arrests (or delays) the progress of embryo development at the brink of radicle growth initiation [10]. After germination, complex biphasic effects of exogenous ABA on root growth were demonstrated [11]. Under well-watered conditions, relatively low concentrations of exogenous ABA maintained the growth of the primary root, while high concentrations inhibited root growth through an ethylene-dependent pathway [11]. These findings are in good agreement with previous studies which have reported that mild soil drying stimulates root growth, but when soil drying becomes more severe, it inhibits root growth [12,13]. Thus, ABA accumulation in drying soil, along with endogenous ABA, plays an important role in maintaining plant water status and growth during water stress [14,15]. However, excessive levels of ABA delay post-stress root growth. In addition, despite the positive effect on the maintenance of the water status of shoots, exogenous ABA can inhibit the emergence of vegetative leaves under non-stress conditions [16] and also cause chlorosis, which accelerates linearly with increasing ABA concentration [16,17]. Moreover, the application of exogenous ABA revealed that increased ABA levels correlated with an increased susceptibility of plants to fungal [18,19,20] and bacterial pathogens [21]. It has been suggested that one of the general functions of ABA is to act as a signaling molecule during inter-species communication [22].

Many bacteria were shown to produce ABA. The ability to synthesize and secrete ABA was found in several plant growth-promoting rhizobacteria (PGPR) including *Achromobacter xylosoxidans* [23,24], *Bacillus subtilis*, *Pseudomonas putida*, *Brevibacterium halotolerans*, *Arthrobacter koreensis* [24], and *Azospirillum baldaniorum* [25]. Inoculation of soil with the ABA-producing bacteria has shown increased resistance of plants to salinity and drought [23,25,26]. ABA production was also shown for some rhizobia [27,28]. However, ABA is generally known to inhibit nodulation [29]. The ability to secrete ABA was described in many phytopathogenic fungi [30] and shown to be an important trait for their virulence [31,32,33]. Exogenous ABA application has been shown to affect the growth and metabolism of the endophytic fungus *Aspergillus nidulans*, inducing vast gene expression changes [34]. A relatively low level of ABA supports the interaction of plants with arbuscular mycorrhizal fungi, while higher concentrations have negative effects on colonization [35]. In this regard, it has been suggested that plant roots excrete ABA to select microbes that are perceptive to this hormone or capable of using it as a carbon source [36].

ABA production has been shown for animals representing various taxonomic groups ranging from sea sponges to mammals [37]. In addition to endogenous biosynthesis, animals also use ABA as a dietary component that may have beneficial physiological effects, including pro-inflammatory actions, cell proliferation stimulations, glucose tolerance, improvement of metabolic and bodily parameters [38,39]. As shown in *Apis mellifera*, in insects, ABA may be involved in wound healing, pesticide sensitivity, and cold resistance [40]. In mice, ABA administration improves neuroinflammation, cognitive impairment and anxiety in rodents [41]. Phaseic acid, the principal ABA metabolite in plants, is endogenously produced in the brain and protects it from ischemic injury [42]. ABA seems to play an important role in mutualistic interactions between animals and the gut microbiome. Despite this, ABA often suppresses host immune responses and is utilized by pathogens as an effector molecule [22].

Most papers on the chemistry of ABA deal with its synthesis [22,43]. It has been shown that plants, fungi, and bacteria have developed different ways of ABA biosynthesis [22]. Meanwhile, catabolism is also very important for ABA homeostasis. In plants, it is known that ABA can be catabolized to phaseic acid via CYP707A or inactivated by glucose conjugation (ABA-glucose ester) via the enzyme uridine diphosphate-glucosyltransferase (UDP-glucosyltransferase) [44]. However, a complete chemical degradation of ABA still awaits development.

Recently, using a selective ABA-supplemented medium, two bacterial strains were isolated from the rhizosphere of rice (*Oryza sativa* L.) and assigned to *Rhodococcus* sp. P1Y and *Novosphingobium* sp. P6W [45]. Both strains could utilize ABA as a sole carbon source in batch culture and decrease ABA concentrations in tomato roots or leaves upon plant inoculation. We have also shown that *Rhodococcus* sp. P1Y is able to shorten the acyl moiety of the ABA molecule to form dehydrovomifoliol [46]. This work is devoted to a further study of ABA catabolism in this bacterial species and presents a new bacterial metabolite.

## 2. Materials and Methods

### 2.1. ABA and Its Tritium Labeled Analog

(+)-ABA was purchased from Merck (Sigma-Aldrich, St. Louis, MO, USA). Tritium was introduced into the cyclohexene part of the molecule as described earlier [47]. The radiochemical purity of the final product was 98.5% and the specific radioactivity was 30.5 Ci mmol^−1^.

### 2.2. Cultivation of the Bacterium

The PGPR strain *Rhodococcus* sp. P1Y was obtained from the Russian Collection of Agricultural Microorganisms (RCAM, St. Petersburg, Russian Federation, http://www.arriam.ru/kollekciya-kul-tur1/ accessed on 25 September 2022). Bacterial culture cells were grown at 24 °C with shaking (200 rpm) in 1000 mL (2 × 500 mL) of a mineral salt medium containing (g L^−1^): 14 Na_2_HPO_4_·12H_2_O, 3.0 KH_2_PO_4_, 1.0 NH_4_Cl, 0.3 MgSO_4_, 0.1 CaCl_2_, pH = 6.5. The medium was supplemented with microelements and 250 mg L^−1^ (+)-ABA. Bacterial growth was monitored by measuring the optical density of suspensions at 540 nm (optical path 1 cm) using a SmartSpec Plus spectrophotometer (Bio-Rad Laboratories Inc., Hercules, CA, USA). When the cell culture absorbance reached 0.4 AU, which corresponded to 18 h after the start of cultivation, an additional 250 mg L^−1^ of ABA and 10 μCi L^−1^ of labeled ABA was added. After 4 h of incubation, the bacterial suspension was centrifuged, and the supernatant was lyophilized.

### 2.3. Sample Extraction and Purification

The lyophilized material was extracted with 150 mL of methanol using an ultrasonic bath. The insoluble residue was collected by centrifugation and extracted with 150 mL of methanol again. The re-extraction procedure was performed twice. The combined extract was evaporated on a rotary evaporator at 40 °C. The resulting residue weighing 507.4 mg was dissolved in 150 mL of 0.1 M NaHCO_3_ and extracted three times in a separation funnel with 100 mL portions of methylene chloride. The aqueous solution was acidified carefully with acetic acid to pH 4–5 and extracted with ethyl acetate three times with 100 mL portions. The combined ethyl acetate solution was dried over Na_2_SO_4_ and evaporated to dryness on a vacuum rotary evaporator Heidolph Hei-VAP Precision (Heidolph Instruments GMBH and CO KG, Schwabach, Germany) at 40 °C. As a result, 461.3 mg of dry yellow residue was obtained.

Preparative chromatographic separation of the residue suspected of containing ABA metabolites was carried out on silica gel (Merck 60) using a Buchi Sepacore MPLC system equipped with a C-630 UV monitor, two C-605 pump modules, an S-620 control unit, and a C-660 fraction collector (Buchi, Flawil, Switzerland). The solvents were (A) *n*-hexane, (B) EtOAc, and (C) MeOH. The dry residue was dissolved in 50 mL of methanol and loaded onto 5 mL of silica gel, blowing off the methanol in a stream of nitrogen. The resulting material was introduced in hexane into a cartridge, which was connected in series with a Glass Column Buchi 15/460 (81.3 mL) filled with the same silica gel and preconditioned in a hexane flow (15 mL min^−1^ for 5 min). The separation conditions are shown in Table 1. The eluent flow rate was 15 mL min^−1^. Fractions of 45 mL were collected and analyzed by liquid scintillation counting using a QuantaSmart Tri-Carb 2810TR instrument (PerkinElmer, Inc., Waltham, MA, USA).

The obtained MPLC fractions were analyzed for their component composition by HPLC using an Acquity class H chromatograph with a PDA detector and a Waters ACQUITY UPLC BEH C18 column (50 × 2.1 mm, 1.7 μm). Chromatography was performed at 30 °C. The injection volume was 5 μL. The flow rate was 0.3 mL min^−1^. The mobile phases were water with 0.1% formic acid (A) and acetonitrile with 0.1% formic acid (B). The gradient was as follows: 0–1 min 0% B, 1–13 min 0% → 90% B; 13–14 min, 90% B; and 14–14.1 min, 90% → 0% B. The detection wavelengths were 265 and 240 nm. Fractions 4–8 contained two compounds with retention times corresponding to ABA and dehydrovomifoliol. Fractions 12–14 contained one unknown compound as the main component with a total content of more than 96%. Fractions 12–14 were combined and evaporated to dryness. Dry residue was dissolved in methanol, recrystallized, and analyzed by HPLC. A total of 155.9 mg (yield 30.7%) of a white crystalline substance was obtained with a chromatographic purity of more than 98%. Identification of the chemical structure of the new ABA metabolite was performed using NMR spectroscopy in combination with mass spectrometry, FTIR spectroscopy, spectrophotometry, and polarimetry.

### 2.4. HPLC-MS Analysis

The substance dissolved in methanol was analyzed by HPLC-MS using a IT-TOF mass spectrometer (Shimadzu Corporation, Kyoto, Japan) equipped with an ESI interface. A Waters BEH-C18 column (50 × 3.0 mm, 3.5 μm, Waters Corporation, Milford, MA, USA) was used. Chromatography was carried out at a temperature +15 °C and a flow rate of 0.3 mL/min. The mobile phases were 0.1% formic acid (A) and acetonitrile +0.1% formic acid (B). The gradient was as follows: 0–1 min 0% B, 1–13 min 0% → 90% B; 13–14 min, 90% B; and 14–14.1 min, 90% → 0% B. The IT-TOF/MS analysis was carried out in full-scan mode, and the mass range was *m*/*z* 60−2000 in the negative and positive modes for MS and MS/MS, with a scan rate 2 spectra/sec. The operating parameters of the electrospray ionization sources were as follows: nebulizing gaz (N_2_) flow rate, 1.5 L/min; drying gas pressure, 100 kPa; CDL temperature, 200 °C; heat block temperature, 200 °C; probe voltage, 3.5 kV for positive mode and –2.5 kV for negative mode; ion accumulation time, 30 ms; collision energy of collision-induced dissociation (CID), 10% for negative mode and 7% for positive mode; collision gas (Ar), 10% for negative and positive mode; detector voltage, 1.6 kV. All the acquisitions and analyses of data were controlled by LabSolutions LCMSSolution software (release 3.80, Shimadzu Corporation). A standard solution of sodium trifluoroacetic acid (TFA) was used to calibrate the TOF–MS to increase mass accuracy. Parallel UV detection was performed using an SPD-M20A Multiple Wavelength Detector instrument (Shimadzu Corporation).

### 2.5. Spectrophotometry

An ultraviolet/visible (UV/Vis) spectrum was recorded in MeOH on a Beckman Coulter DU 800 spectrometer (Beckman Coulter Inc., Brea, CA, USA). The survey was carried out in a quartz cuvette with an optical path length of 1 cm. Survey parameters: measurement range 200–800 nm, resolution 0.1 nm, scanning speed 120 nm/min. The concentration of the compound was 0.01 mol/L.

### 2.6. Polarimetry

Optical rotation was determined using an automatic polarimeter SAC-i (ATAGO Co., Ltd., Tokyo, Japan) for a monochromatic laser beam with λ = 589 nm. The cell length (optical path) was 50 mm. For measurement, a solution of the substance in water with a concentration of 20 mg/mL was used. The measurement was carried out at room temperature (22.3 °C). The resulting value is the average of 20 measurements.

### 2.7. Melting Point Measurement

The melting point was determined on a PTP-M instrument (Khimlabpribor, Ltd., Klin, Russia) according to the manufacturer’s manual.

### 2.8. FTIR Spectroscopic Measurements

For FTIR spectroscopic measurements, a sample of the isolated and purified compound (~1 mg) was dissolved in a minimal volume (~10 μL) of MilliQ water, placed (using a microsyringe) as a thin film on a clean flat ZnSe disk (CVD-ZnSe, “R’AIN Optics”, Dzerzhinsk, Russia; ø 1 cm, thickness 0.2 cm) and dried in a drying cabinet at 45 °C (~20 h). Transmission FTIR spectroscopic measurements were performed on a Nicolet 6700 FTIR spectrometer (Thermo Electron Corporation, Beverly, MA, USA; DTGS detector; KBr beam splitter) as reported earlier [46] (with a total of 64 scans (resolution 4 cm^−1^) against the ZnSe disk background; spectra were manipulated using the OMNIC software (version 8.2.0.387) supplied by the manufacturer of the spectrometer). The baseline was corrected using the “automatic baseline correct” function, and the spectra were smoothed using the standard “automatic smooth” function of the software which uses the Savitsky–Golay algorithm (95-point moving second-degree polynomial). The FTIR spectroscopic measurements were repeated three times and were well reproducible.

### 2.9. NMR Spectroscopic Measurements and Experimental Conditions

One-dimensional (^1^H, ^13^C) and two-dimensional (HSQC, HMBC, H2BC) NMR spectra were recorded on a DirectDrive NMR System (Varian, Palo Aho, CA, USA) in D_2_O at 700 and/or 175.8 MHz. The residual signals of H_2_O (for ^1^H NMR), as well as of CH_3_OH (for ^13^C NMR) which was introduced as a micro-impurity in one of the experiments, were used as internal standards.

## 3. Results

### 3.1. Isolation of Metabolite 2

**Figure 1:**
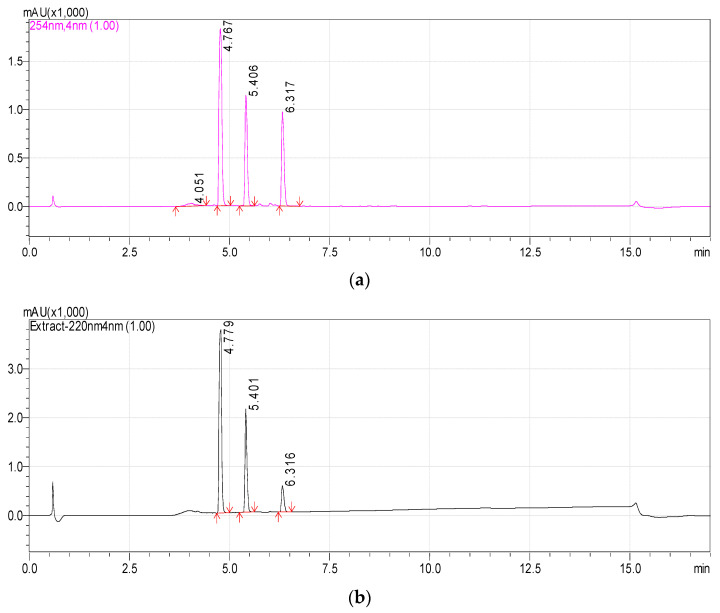
Chromatograms of the components of the *Rhodococcus* sp. P1Y culture liquid 4 h after the tritium labeled ABA was added (methanol solution). HPLC-MS, registration at two wavelengths: 254 nm (**a**) and 220 nm (**b**). Main components: Metabolite 2 (RT 4.8 min), Dehydrovomifoliol (RT 5.4 min), ABA (RT 6.3 min).

**Figure 2:**
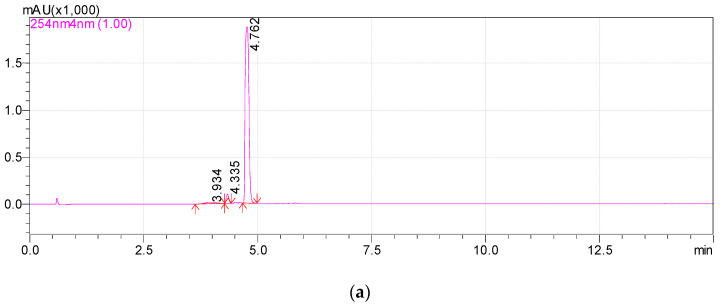

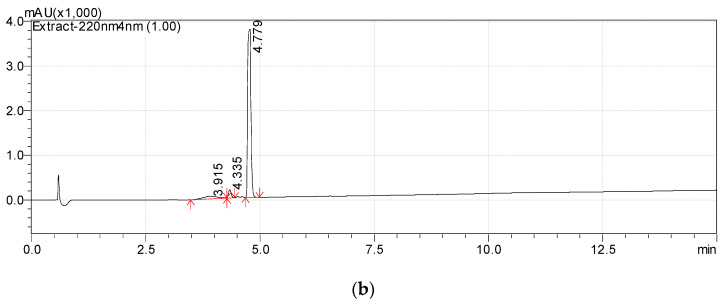
Chromatograms of Metabolite 2 (RT 4.8 min) in methanol solution. HPLC-MS, registration at two wavelengths: 254 nm (**a**) and 220 nm (**b**).

Previously, we reported that the growth of *Rhodococcus* sp. P1Y on the minimal medium with ABA was accompanied by the degradation of this phytohormone and the accumulation of additional metabolites in the culture liquid. HPLC-MS revealed two putative metabolites, one of which was purified and identified as dehydrovomifoliol [46]. In the present work, we increased the cultivation time after the addition of ABA from 2 to 4 h, which increased the accumulation of the second compound (Figure 1, compound with a retention time (RT) of 4.8 min) in the culture liquid with a simultaneous decrease in the content of dehydrovomifoliol and ABA (Figure 1, compounds with RT of 5.4 and 6.3 min, respectively). Using the procedures described in Section 2.3 allowed us to purify Compound 2 to an individual form (over 98 percent, Figure 2).

The monoisotopic molecular weight of this compound, according to the results of mass spectrometry (see Section 3.2), was 212.1049 Da. This corresponded to the molecular formula C_11_H_16_O_4_. Bacteria were cultivated using radioactive ABA with a calculated specific radioactivity of 10.57 μCi mmol^−1^ (10 μCi per 250 mg of ABA with a molecular weight of 264.32 Da). The individual compound had a specific radioactivity of 9.12 μCi mmol^−1^ (9.55 × 10^4^ cpm m^−1^). It should be noted that some discrepancy between the values may be due to the fact that the accumulation of bacterial biomass was carried out in preliminary cultivation using non-radioactive ABA. Probably, a slight overestimation of the specific radioactivity of the starting material can be caused by residual amounts of ABA in the culture medium before the addition of the radioactive preparation. Thus, the initial substrate and the purified compound have a comparable content of the radioactive isotope. This may serve as evidence that the isolated compound is an ABA derivative. Further, this substance was designated “Metabolite 2”. The use of a complex of physicochemical methods and instrumental techniques described below made it possible to identify Metabolite 2 as 1-hydroxy-2,6,6-trimethyl-4-oxo-2-cyclohexene-1-acetic acid.

**1-hydroxy-2,6,6-trimethyl-4-oxo-2-cyclohexene-1-acetic acid** (**Metabolite 2**). mp: 118–120 °C (decomp.). [∝]${}_{D}^{20}$ = +33.6° (Δ = 0.6°). UV: λ_max_, nm (log ε): 240.6 (2.23). IR (ν_max_, cm^−1^; band shape and vibration type assignments are given in parentheses): 3600–3100 (very broad, asymmetric; ν OH in H-bonded OH and COOH); 3028 (weak; ν =C–H); 2963, 2933, 2917, 2876, 2852 (ν_as_ CH_3_, ν_as_ CH_2_, ν_s_ CH_3_, ν_s_ CH_2_); 1707 (asymmetric, broad; ν C=O), 1658 (ν C=C), 1465 (shoulder), 1454 (δ_as_ CH_3_, scissoring δ CH_2_), 1593, 1399 (strong, broad; ν_as_ and ν_s_ in –COOH); 1281 (in-plane δ(C–O–H) in COOH). ^1^H NMR (ppm): 5.81 (1H, s, H-3′), 2.53 (1H, d, J = 15.1 Hz, H-2b), 2.48 (1H, d, J = 17.9 Hz, H-5′a), 2.32 (1H, d, J = 15.1 Hz, H-2a), 2.18 (1H, d, J = 17.9 Hz, H-5′b), 1.92 (3H, s, H-7′), 0.95 (3H, s, H-8′), 0.90 (3H, s, H-9′). ^13^C NMR (ppm): 205.7 (C-4′, C=O), 183.0 (C-1, C=O), 174.0 (C-2′, Cq), 127.7 (C-3′, HC=C), 79.9 (C-1′, C–OH), 51.5 (C-5′, CH_2_), 44.2 (C-6′, Cq), 42.4 (C-2, CH_2_), 25.7 (C-8′, CH_3_), 25.2 (C-9′, CH_3_), 22.3 (C-7′, CH_3_). MS (MS/MS), *m*/*z* (relative intensity): Negative ion mode, no collision: 212.1003 (^13^C-Isotope ion, calcd. 212.1004) [M–H^+^ + 1]^−^ (12), 211.0960 (C_11_H_15_O_4_, calcd. 211.0970) [M–H^+^]^−^ (100), 167.1062 (C_10_H_15_O_2_, calcd. 167.1072) [M–H^+^–CO_2_]^−^ (2), 149.0985 (C_10_H_13_O, calcd. 149.0966) [M–H^+^–CO_2_–H_2_O]^−^ (1), 112.9886 (C_4_HO_4_, calcd. 112.9875) (38); Negative ion mode, collision energy of CID 10%, molecular ion 211.0970 (ΔM = 1.0000) fragmentation: 211.0959 (C_11_H_15_O_4_, calcd. 211.0970) [M–H^+^]^−^ (100), 167.1088 (C_10_H_15_O_2_, calcd. 167.1072) [M–H–CO_2_]^−^ (91), 152.0852 (C_9_H_12_O_2_, calcd. 152.0837) [M–H^+^–CH_3_COOH]^−^ (6), 111.0484 (C_6_H_7_O_2_, calcd. 111.0446) (3); Positive ion mode, no collision: 214.1174 (^13^C-Isotope ion, calcd. 214.1161) [M + H^+^ + 1]^+^ (13), 213.1133 (C_11_H_17_O_4_, calcd. 213.1127) [M + H^+^]^+^ (100), 195.1024 (C_11_H_15_O_3_, calcd. 195.1021) [M + H^+^ − H_2_O]^+^ (46), 149.0997 (C_10_H_13_O, calcd. 149.0966) [M + H^+^ − H_2_O − HCOOH]^+^ (46), 135.0840 (C_9_H_11_O, calcd. 135.0810) [M + H^+^ − H_2_O − CH_3_COOH]^+^ (6), 129.0576 (C_6_H_9_O_3_, calcd. 129.0552) (6), 105.0508 (C_4_H_9_O_3_, calcd. 105.0552) (7), 91.0565 (C_7_H_7_, calcd. 91.0548) (8); Positive ion mode, collision energy of CID 7%, molecular ion 213.1133 (ΔM = 1.0000) fragmentation: 213.1103 (C_11_H_17_O_4_, calcd. 213.1127) [M + H^+^]^+^ (100), 195.1018 (C_11_H_15_O_3_, calcd. 195.1021) [M + H^+^− H_2_O]^+^ (56).

### 3.2. Mass Spectrometric Studies

**Figure 3:**
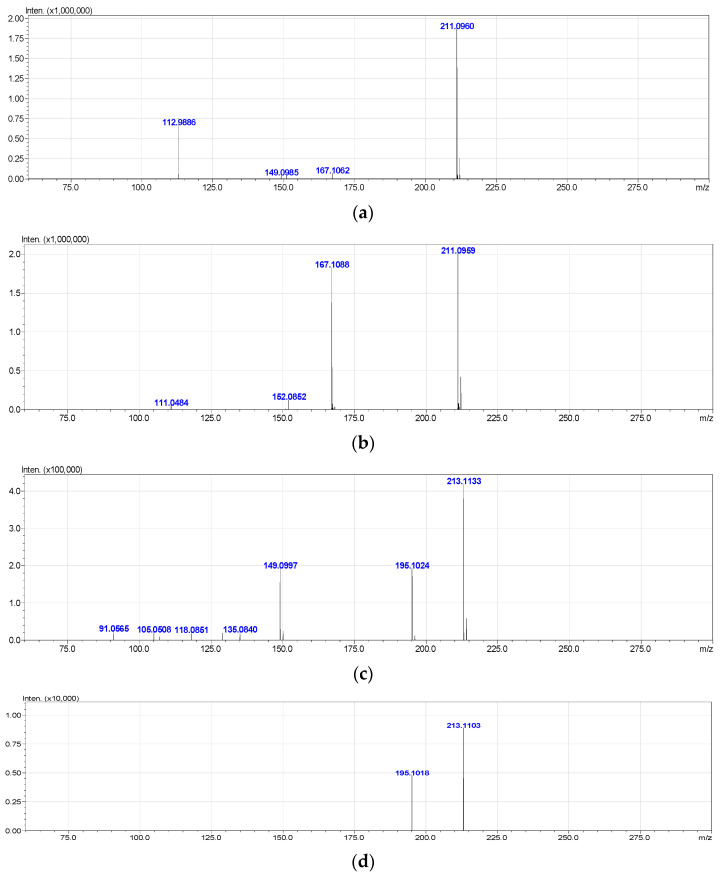
Mass spectra of Metabolite 2 (ESI–TOF MS); (**a**) negative ion mode, no collision; (**b**) negative ion mode, collision energy of CID 10%, molecular ion 211.0972 (ΔM = 1.0000) fragmentation; (**c**) positive ion mode, no collision; (**d**) positive ion mode, collision energy of CID 7%, molecular ion 213.1133 (ΔM = 1.0000) fragmentation.

The high resolution mass spectra of Metabolite 2 obtained in the negative and positive electrospray modes are shown in Figure 3. The interpretation of these spectra gives insight into the structure of the metabolite. The generation of a stable negatively charged molecular ion 211.0960 (calculated 211.0970) under experimental conditions indicates the acidity of the compound. The 167.1062 (calculated 167.1072) ion associated with the elimination of the neutral CO_2_ molecule may indicate the presence of a carboxyl group in its molecular structure. The same assumption indirectly confirms the elimination of the CH_3_COOH molecule. The splitting off of a water molecule from a molecular ion obtained in the positive ionization mode suggests the presence of a hydroxyl group in the compound under study.

**Figure 4:**
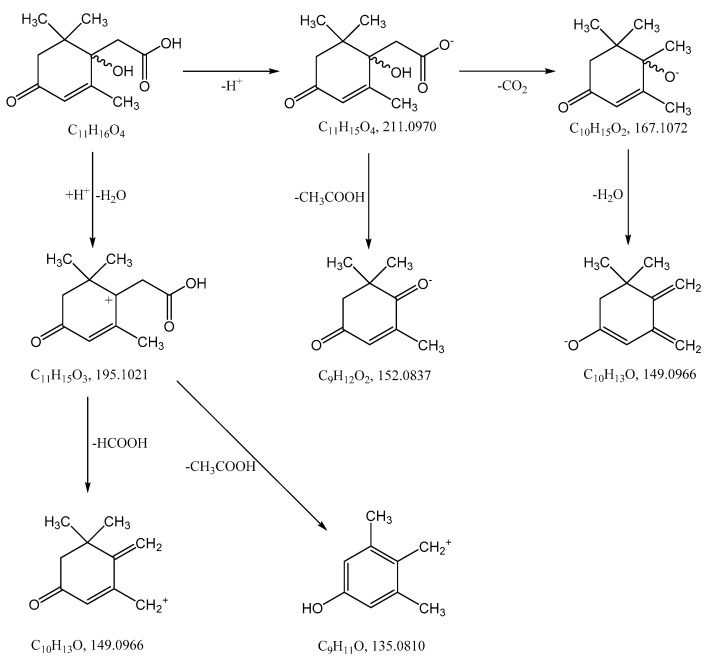
Hypothetical structure and scheme of fragmentation of Metabolite 2 molecular ions under negative and positive electrospray ionization conditions.

The most probable molecular formula for the 211.0960 ion is C_11_H_15_O_4_, for the 213.1133 ion it is C_11_H_17_O_4_. Along with the structure and known pathways of fragmentation of the initial ABA molecule, this allows us to suggest the most probable structure of the metabolite and the fragmentation scheme of its molecular ions, which are shown in Figure 4.

### 3.3. NMR Spectroscopy

**Figure 5:**
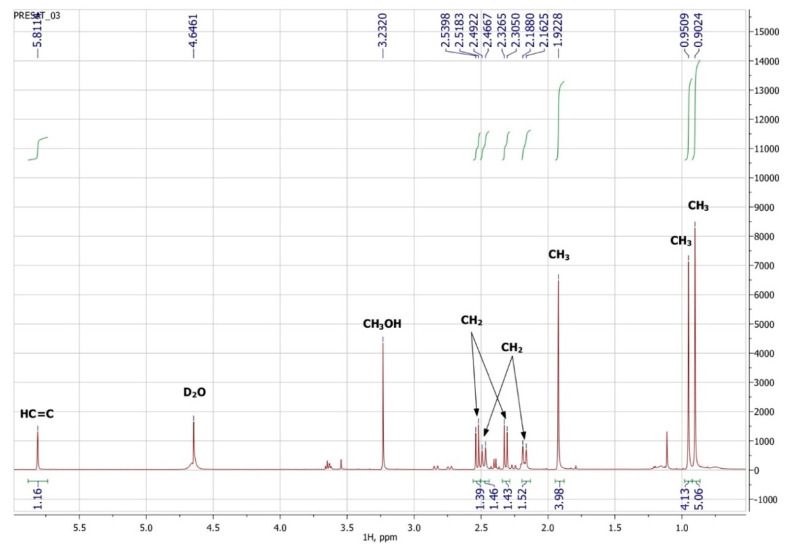
^1^H NMR spectrum of Metabolite 2 (D_2_O, operating frequency 700 MHz).

**Figure 6:**
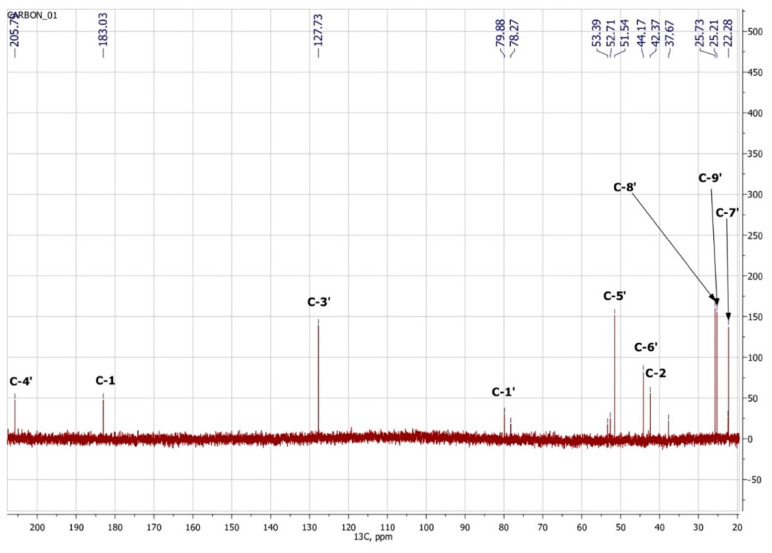
^13^C NMR spectrum of Metabolite 2 (D_2_O, operating frequency 175.8 MHz).

**Figure 7:**
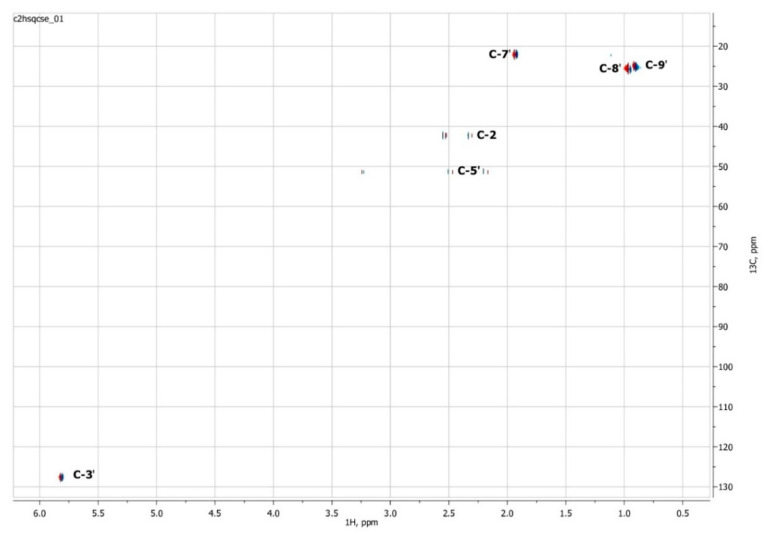
Two-dimensional ^13^C–^1^H HSQC NMR spectrum of Metabolite 2, obtained in D_2_O at 700 MHz for ^1^H and 175.8 MHz for ^13^C.

**Figure 8:**
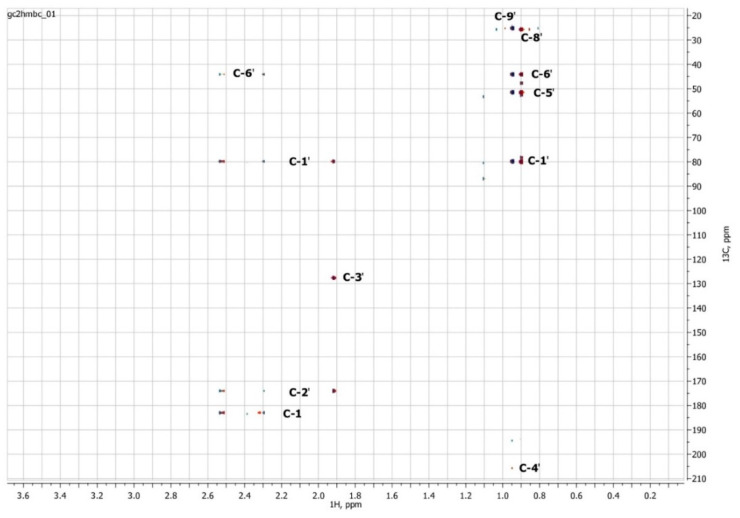
Two-dimensional ^13^C–^1^H HMBC NMR spectrum of Metabolite 2, obtained in D_2_O at 700 MHz for ^1^H and 175.8 MHz for ^13^C.

To confirm the presented hypothetical structure of the Metabolite 2 molecule, we performed one-dimensional (^1^H, ^13^C) and two-dimensional (^13^C–^1^H HSQC, ^13^C–^1^H HMBC, H2BC) NMR experiments. The results are shown in Figure 5, Figure 6, Figure 7 and Figure 8, respectively. The original NMR spectral characteristics of Metabolite 2 are presented in Table 2.

**Figure 9:**
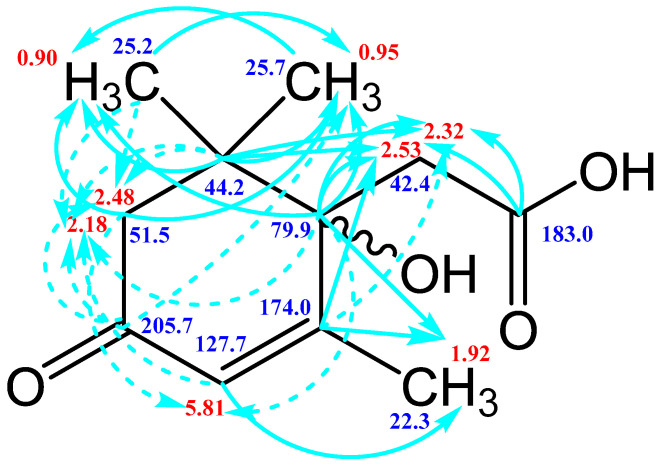
Scheme of long-range spin-spin interactions ^13^C–^1^H in the Metabolite 2 molecule, constructed basing on the analysis of two-dimensional spectra of HSQC, HMBC, and H2BC. Each arrow starts at a carbon atom and ends at a proton. The dotted line denotes weak interactions.

The HSQC spectrum made it possible to establish the binding of carbon atoms to the corresponding protons, as well as to determine the nature of atomic groups. Information about the carbon skeleton of the molecule was obtained from the two-dimensional spectra of long-range spin-spin interaction. The proton spin-spin coupling constants were extracted from the ^1^H spectrum. The absence of the proton peak of the tertiary hydroxyl group in the ^1^H NMR spectrum can be explained by the presence of a stable intramolecular hydrogen bond with the oxygen of the carboxyl group of the side chain (see the FTIR spectroscopic data). The long-range ^13^C–^1^H interaction constants are schematically shown in Figure 9. The great general similarity of the NMR spectra of Metabolite 2 with those for abscisic acid and dehydrovomifoliol should be emphasized, especially for the cyclic part of the molecule [46], which confirms the common chemical nature of all the three compounds. In fact, all spectra of Metabolite 2 are a reduced version of the spectra of ABA and dehydrovomifoliol, which, apparently, may indicate the origin of this compound from more complex precursors. Summarizing the presented results, we can conclude that, according to the spectral data obtained both in NMR experiments and by mass spectrometry and optical spectrometry, the cyclic part of the molecule in Metabolite 2 is similar to that of original ABA. The structure of the side chain of a molecule can be established by considering its NMR spectra as a whole. The presence of a cross peak in the HMBC spectrum between the carbon atom of the carboxyl group (δ = 183.0) and two protons of the CH_2_ group (δ = 2.32, 2.53) indicates the presence of bonding between carbon atoms with δ = 183.0 and δ = 42.4. Similarly, the interaction of a quaternary carbon atom at δ = 79.9 with the above protons indicates the presence of an interaction between carbon atoms with δ = 79.9 and δ = 42.4. Thus, the presented data, in our opinion, are sufficient to establish the structure of the side chain and the position of its attachment to the cyclic part of the molecule.

**Figure 10:**
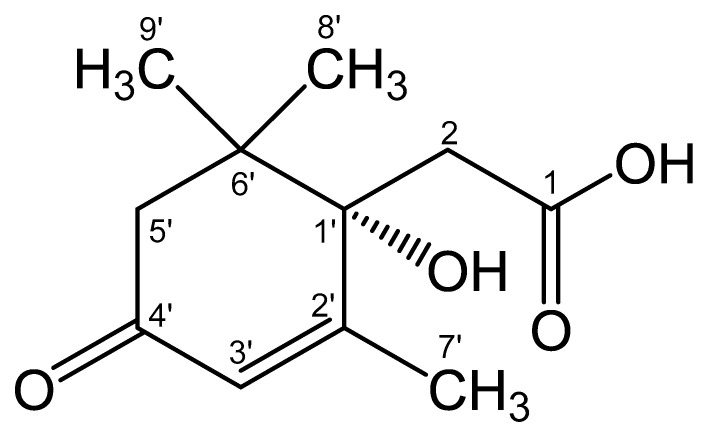
Structural formula of the isolated compound defined from the results of spectroscopic characterization as 1-hydroxy-2,6,6-trimethyl-4-oxo-2-cyclohexene-1-acetic acid. The name of the compound corresponds to the cyclohexene carbon atoms C1′–C6′ (in the Figure) numbered as C1–C6, respectively. The whole numbering shown in the Figure is for the purpose of discussions only. The hydroxyl configuration at C1′ is preliminarily based on the studies discussed in Section 3.6.

The results obtained allowed us to establish the structure of Metabolite 2 as 1-hydroxy-2,6,6-trimethyl-4-oxo-2-cyclohexene-1-acetic acid (Figure 10). To the best of our knowledge, this substance has not been previously described. This compound was given the trivial name rhodococcal acid (RA). Additional information about the structure of this compound was obtained by optical spectroscopy.

### 3.4. UV-Visible Absorption Spectrum of Metabolite 2

**Figure 11:**
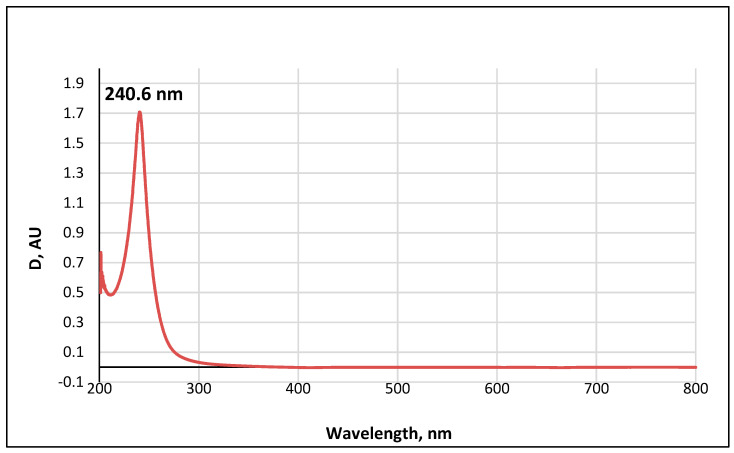
UV-Vis absorption spectrum of a 0.01 M methanolic solution of ABA metabolite 2.

The UV-visible spectrum of a 0.01 M methanolic solution of the ABA metabolite (Figure 11) shows a single band with an absorption wavelength λ_max_ = 240.6 nm.

**Figure 12:**
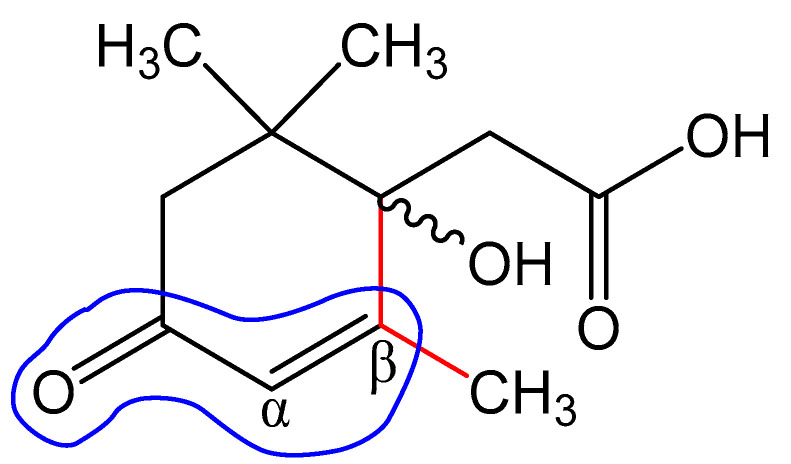
Structural elements of abscisic acid Metabolite 2 that contribute to the calculation of the maximum absorption wavelength according to Woodward’s rules.

The obtained value correlates well with the wavelength calculated according to Woodward’s empirical rules [48] for π→π* transitions in conjugated enone systems (Figure 12): λ_max_ (calculated) = λ_0_ + 2 × Δλalk_β_ = 215 + 2 × 12 = 239 nm. The base value λ_0_ = 215 nm was taken to be the absorption maximum for cyclic enones in six-carbon cycles. It should be noted that dehydrovomifoliol, which is also a degradation product of abscisic acid, has a similar spectrum with λ_max_ = 239 nm [46].

### 3.5. FTIR Spectroscopic Characterization

**Figure 13:**
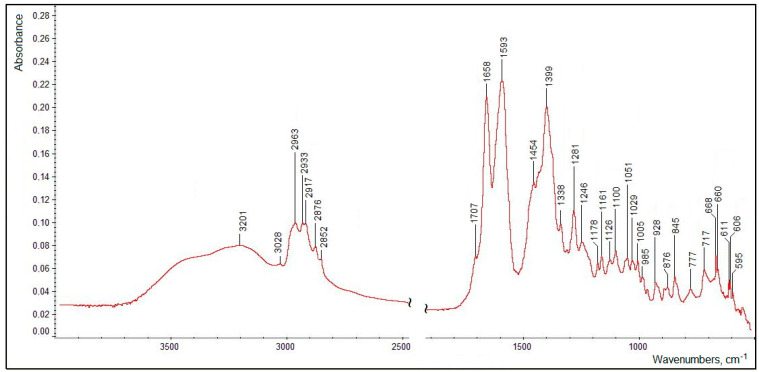
A FTIR spectrum of the isolated and purified title compound (measured in the transmission mode).

The FTIR spectrum of the isolated and purified title compound (measured as a dry thin film of the pure substance, without a solvent or a matrix) presented in Figure 13 clearly features all the functional groups defined in its structure (see Figure 10). Assignments of the main characteristic bands observed in the FTIR spectrum of the title compound are given in Table 3.

The very broad ν(OH) region evidently contains two poorly resolved broadened maxima corresponding both to the tertiary alcoholic hydroxo group (at C1′; see Figure 10) at ~3500–3400 cm^−1^ (which was observed for dehydrovomifoliol, with a similar structure but lacking the carboxylic group, at 3448 cm^−1^ [46]) and to the carboxylic OH-group (with a maximum at 3201 cm^−1^). It is natural that in the condensed phase both of these moieties are involved in relatively strong intermolecular H-bonding (see the description of the ^1^H NMR data presented above).

The ν(C–H) region comprises a number of bands (see Table 3) typical of methyl and methylene groups; note also the weak ν(=C–H) band (for the group at the C3′ position) at 3028 cm^−1^ and the slight difference in the frequencies of the ν_as_(CH_2_) bands for the side chain (position C2) and in the cycle (position C5′) neighboring the carbonylic group at C4′. The latter, considering its relatively low frequency (1707 cm^−1^) as compared to that in dehydrovomifoliol (1736 cm^−1^ [46]), is also involved in the intermolecular H-bonding. The positions of the ν(C=C) band (1658 cm^−1^), stretching vibrations of the carboxylic group and of a number of bending modes (see Table 3) are characteristic; the lower-wavenumber region of the spectrum in Figure 13 contains a number of weak bending and stretching bands of the C–C/C–O skeleton which are less specific.

Thus, the main set of characteristic bands listed in Table 3 (see Figure 13) can be useful for the identification of this substance by spectroscopic techniques including FTIR spectroscopy.

### 3.6. Polarimetry

An aqueous solution of Metabolite 2 showed the presence of optical rotation: [∝]${}_{D}^{20}$ = +33.6° (Δ = 0.6°). This clearly indicates the absence of racemization during the biodegradation of the side chain of the ABA molecule. The fact that the isolated substance is not racemic indicates the enzymatic nature of the observed conversion of ABA. Probably, the position of the hydroxyl group at carbon atom 1′ of RA coincides with that for ABA, as shown in Figure 10.

### 3.7. Melting Point Measurement

**Figure 14:**
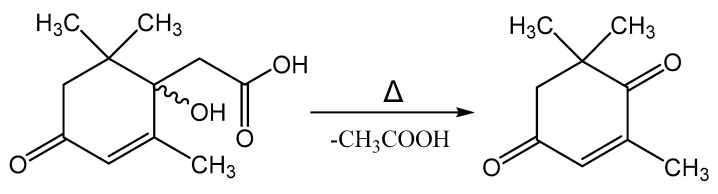
Decomposition reaction of Metabolite 2 on heating.

Melting of Metabolite 2 is accompanied by its decomposition. At a temperature of 118–120 °C, a gaseous product is released and the substance passes into a liquid state. Further heating of the substance leads to its evaporation in the temperature range of 220–225 °C. Mass-spectrometric analysis of the compound after the decomposition process by GC-MS allowed it to be identified as 4-oxoisophorone. Thus, the process of decomposition of Metabolite 2 is apparently associated with the elimination of an acetic acid molecule, as shown in Figure 14.

## 4. Discussion

**Figure 15:**
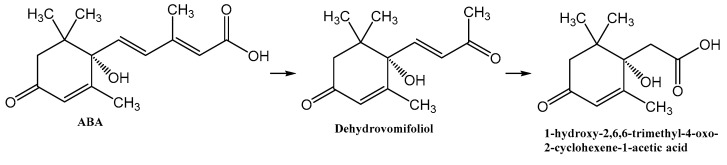
Confirmed steps of the initial stage of microbial catabolism of ABA.

ABA is well studied as a phytohormone [1,15]. However, the discovery of this compound in a wide range of organisms, including bacteria, algae, fungi, sea sponges and mammals, determined the expansion of the scope of research into the metabolism and functions of ABA [22,37,50]. At the same time, most reports on the chemistry of ABA deal with its synthesis [51]. As a result of these studies, it was shown that plants and fungi have evolved different synthesis pathways using carotenoids (either farnesyl diphosphate or farnesyl pyrophosphate) as precursors [52]. Different prokaryotic species probably use independently developed pathways for ABA biosynthesis, carotenoid-dependent and carotenoid-independent [22]. As regards ABA catabolism, it is known that, in addition to the formation of conjugates, mainly with glucose, plants are limited to the modification of the cyclohexene part of the molecule [51,53,54], while no pathways for complete degradation of the carbon skeleton have been found [55]. Another aspect of the problem is that, as discussed in Section 2, the plant moisture monitoring strategies and plant-microbe communication may involve secretion of ABA into the soil via root exudation [6,36]. In addition, ABA is constantly introduced into the soil with dead tissues and falling leaves and accumulates as a result of production by bacteria, fungi, and algae [6]. Despite the significant biological activity of exogenous ABA [2,11,12], plants do not have the ability to control the soil concentration of this phytohormone or otherwise reduce its activity in the environment. It is likely that in the course of evolution, plants delegated this function to the microorganisms associated with them. However, information on the mechanisms of microbial ABA catabolism is very limited [31,37]. It was reported that the introduction of radioactive ABA into non-sterile soil led to the decomposition of this compound to phaseic acid and dehydrophaseic acid [6]. It was also shown that *Corynebacterium* sp., growing on an ABA-supplemented medium, accumulated a compound with spectral characteristics close to those for dehydrovomifoliol [56]. We have recently isolated this substance from a culture of *Rhodococcus* sp. P1Y in vitro and characterized it in detail as a metabolite of ABA [46]. In the present study, it was shown that *Rhodococcus* sp. P1Y carries out further destruction of ABA, shortening the side chain by two more carbon units. Thus, the first steps of ABA microbial catabolism differ from the known transformations of this phytohormone in plants. This is probably due to the fact that it is important for plants to modify the signaling properties of ABA, while for bacteria this compound is a nutrient source of carbon, for which the use of C_2_ units of the side chain is preferable. The results obtained allowed us to suggest a catabolic pathway for the utilization of the ABA side chain (Figure 15). It can be assumed that in each stage of this catabolic pathway, one molecule of acetate is formed, which is used for the metabolism of bacteria. We believe that to date we have only been able to isolate the initial stages of this catabolic pathway.

At present, the question on the mechanisms of the presented initial stages of ABA degradation and the enzymes involved in them has yet to be developed. The transcriptome data showed that in *Rhodococcus* sp. P1Y (data not published) and *Novosphingobium* sp. P6W [57], the growth of bacteria on a medium with ABA increases the level of fatty acid metabolism, the enzymes of which can be involved in ABA utilization. In this regard, it should be noted that the branch point at the C3 position of ABA may interfere with normal β-oxidation. However, it was previously shown that *Rhodococcus ruber* [58] and *Nocardia cyriacigeorgica* [59] can overcome similar branching points of aliphatic and aromatic hydrocarbons, in particular, they can shorten β-methylcinnamic acid by cleaving the C2-unit to form acetophenone. Probably, *Rhodococcus* sp. P1Y can implement similar mechanisms in relation to ABA during the formation of dehydrovomifoliol. The formation of RA from dehydrovomifoliol can occur as a result of subterminal oxidation with the participation of Baeyer-Villiger monooxygenase, accompanied by the accumulation of acetate [60]. However, additional studies are required to accurately establish the mechanisms of ABA destruction by soil bacteria. Another limitation of this study is that we were unable to determine the exact stereochemical configuration of the new compound. The configuration shown in Figure 10 is based on indirect information, and an unambiguous conclusion could be drawn from the results of future crystallographic studies.

Regardless of the results of biochemical studies, there are reasons to believe that ABA-utilizing bacteria perform an important ecological function of maintaining soil hormonal homeostasis, normalizing the regulation of plant growth, development, and fitness. Since the production of ABA is a virulence factor for many phytopathogens, in particular, phytopathogenic fungi [32], it may be speculated that ABA-degrading bacteria can possess a biocontrol trait, which makes them a promising object for application in agriculture. It is possible that in other ecological niches, such as the digestive tract of phytophages, microbial degradation of ABA may affect interspecies interactions. Thus, a new pathway for ABA biodegradation may become another interesting aspect of the study of the microbiome. The biological activity of the new isolated compound also needs to be studied.

## 5. Conclusions

In a culture of the rhizospheric bacterium *Rhodococcus* sp. P1Y grown in the presence of radioactive ABA, a new metabolite of this phytohormone was found. This metabolite has been identified as a previously unknown compound, 1-hydroxy-2,6,6-trimethyl-4-oxo-2-cyclohexene-1-acetic acid, for which the trivial name rhodococcal acid has been proposed. Basing on the structural formula, rhodococcal acid is a product of a new metabolic pathway of ABA degradation. We have described the beginning of this pathway, which consists in the sequential cleavage of the side chain of the ABA molecule with the formation of dehydrovomifoliol and rhodococcal acid. Further studies can be aimed at searching for and identifying the subsequent products of this pathway, elucidating the chemical reactions and enzymes involved in their catalysis, characterizing the biological properties of the new compound, and the place of ABA-degrading bacteria in interspecies interactions.

## Figures and Tables

**Table 1:** Conditions for MPLC separation of metabolites and radioactivity of chromatographic fractions of the *Rhodococcus* sp. P1Y culture after incubation for 2 h in the presence of ^3^H-labeled ABA.

No. of Fraction ^1^	Starting Solvents Ratio, vol.%	Final Solvents Ratio, vol.%
1	A 100	A 100
2	A 100	A-B 50:50
3	A-B 50:50	A-B 50:50
4	A-B 50:50	B 100
5–7	B 100	B 100
8	B 100	B-C 80:20
9–11	B-C 80:20	B-C 80:20
12	B-C 80:20	B-C 60:40
13	B-C 60:40	B-C 60:40
14	B-C 60:40	B-C 40:60
15–17	B-C 40:60	B-C 40:60
18	B-C 40:60	B-C 20:80
19	B-C 20:80	B-C 20:80
20	B-C 20:80	C 100
21, 22	C 100	C 100

^1^ The volume of each fraction was 45 mL.

**Table 2:** Data for ^1^H and ^13^C NMR spectra of Metabolite 2 in D_2_O obtained at 700 MHz for ^1^H and 175.8 MHz for ^13^C.

Atom Number	^1^H				^13^C		Group
	δ, ppm	J, Hz	m	nH	δ, ppm	nC	
4′					205.7	1	C=O
1					183.0	1	C(O)OH
2′					174.0	1	Cq
3′	5.81		s	1	127.7	1	HC=C
1′					79.9	1	C–OH
5′a	2.48	17.9	d	1	51.5	1	CH_2_
5′b	2.18	17.9	d	1			
6′					44.2	1	Cq
2a	2.32	15.1	d	1	42.4	1	CH_2_
2b	2.53	15.1	d	1			
8′	0.95		s	3	25.7		CH_3_
9′	0.90		s	3	25.2	1	CH_3_
7′	1.92		s	3	22.3	1	CH_3_

**Table 3:** Assignments of the main characteristic bands in the FTIR spectrum of the isolated and purified title compound [46,49].

Band Maxima (Wavenumber Region), cm^−1^	Assignments ^a,b^
3201, v.br. (~3600–3100)	ν(OH) in H-bonded hydroxo- and carboxylic groups
3028	ν(=C–H) at C3′
2963	ν_as_(CH_3_)
2933	ν_as_(CH_2_), C2
2917	ν_as_(CH_2_), C5′
2876	ν_s_(CH_3_)
2852	ν_s_(CH_2_)
1707	ν(C=O), C4′
1658	ν(C=C)
1593, br.	ν_as_(COO), carboxylic group
1454	δ_as_(CH_3_)
~1465, sh.	δ(CH_2_), scissoring
1399, br.	ν_s_(COO), carboxylic group
~1372, sh.	δ_s_(CH_3_)
1338	δ(–CH=), C5′
1281	δ(C–O–H), in-plane (carboxylic group)

^a^ Abbreviations: ν, stretching; ν_as_, antisymmetric stretching; ν_s_, symmetric stretching; δ, bending; δ_as_, antisymmetric bending; δ_s_, symmetric bending; br., broadened; sh., shoulder; v.br., very broad. ^b^ For positions of carbon atoms or functional groups, see Figure 10.

## Data Availability

All relevant data are within the paper.
